# Betaine supplementation during pregnancy improves postpartum depression, reflexive motor behaviors and brain tissue antioxidant levels in mice offspring

**DOI:** 10.1016/j.crphys.2026.100196

**Published:** 2026-11-07

**Authors:** Negin-sadat Zargarian, Shahin Hassanpour, Razieh Hosseini

**Affiliations:** a Faculty of Veterinary Medicine, SR.C., Islamic Azad University, Tehran, Iran; b Department of Veterinary Basic Sciences, SR.C., Islamic Azad University, Tehran, Iran

**Keywords:** Betaine, Morphine withdrawal syndrome, Postpartum depression, Reflexive motor behaviors, Mice

## Abstract

This study sought to assess the protective impacts of betaine during pregnancy on postpartum depression, reflexive motor behaviors, and antioxidant levels in the offspring of mice experiencing morphine withdrawal syndrome. Group 1 was maintained as the control. The second group included male and female mice that had refrained from morphine, both with no prior exposure to illicit drugs; the third group consisted of morphine-abstinent female mice and male mice without any history of morphine. The fourth group included both male and female mice that had never received drugs. Groups 5-7 resembled groups 2-4, which received betaine (30 mg/kg) administered to the mice treated with morphine. After delivery, post-partum depressive behaviors were assessed using the tail suspension test (TST) and the forced swimming test (FST). After delivery, reflexive motor activity was assessed in pups from each group. Additionally, blood and brain tissue samples were obtained from both the mother and the pups to assess antioxidant activity. The results indicated that reflexive motor behaviors showed a statistically significant decrease following prenatal morphine exposure (P < 0.05). The administration of betaine and morphine considerably enhanced reflexive motor activities in mouse offspring (P < 0.05). Morphine treatment notably elevated post-partum immobility duration in TST and FST (P < 0.05), while betaine treatment significantly reduced immobility duration in TST and FST (P < 0.05). The administration of morphine markedly increased malondialdehyde (MDA) levels while reducing dismutase (SOD), glutathione peroxidase (GPx), and catalase (CAT) levels in the serum of mothers and the brain tissue of the pups, respectively (P < 0.05). The administration of betaine decreased MDA and enhanced SOD, GPx and CAT levels in serum of the mothers and brain tissue of the pups, respectively (P < 0.05). These findings suggested maternal exposure to betaine seems to offer protection against the negative impacts of morphine on post-partum depression and reflexive motor behaviors in the mothers and pups.

## Highlights

• Findings suggested maternal exposure to betaine seems to offer protection against the negative impacts of morphine on post-partum depression and reflexive motor behaviors in the mothers and pups.

## 1 Introduction

The misuse of morphine is a significant medical and social issue globally. Frequent utilization of morphine leads to the emergence of dependency, restricting its application for long-term pain. Morphine addiction is a compulsive behavior involving the pursuit and consumption of the drug, driven by the positive reinforcement of its pleasurable effects and the negative reinforcement of withdrawal symptoms that occur when use is stopped (Luo et al., 2012). While it is established that anatomical structures such as the ventral tegmental area, nucleus accumbens, prefrontal cortex, amygdala, and hippocampus are linked to morphine dependence, the cellular and molecular processes involved are still largely unclear (Stevens and Mohan, 2021). So far, there have been limited effective measures to address morphine dependence (Bedard et al., 2023). Long-term use of morphine leads to a dependent condition marked by neuroadaptive intracellular alterations in response to a modified pharmacological state. Abrupt or drug-induced cessation of long-term opiate use leads to numerous physical and mental withdrawal symptoms due to these changes in the nervous system (Farhadi et al., 2024). Growing evidence indicates that males exhibit greater sensitivity than females to the symptoms of morphine withdrawal syndrome. While the fundamental mechanisms behind these sex differences remain unclear, it has been proposed that alterations in μ-opioid binding sites might explain this sexual dimorphism (Molladizavandi et al., 2023).

The postpartum period is a time of fragility as women encounter various stressors. For women struggling with opioid use disorder, the interaction of these ‘Fourth Trimester’ stressors combined with addiction-related issues may lead to overdose becoming a primary cause of postpartum deaths (Baldo, 2022). Additionally, treating an infant for neonatal opioid withdrawal syndrome may be linked to a higher risk of postpartum overdose, especially for women undergoing treatment for opioid use disorder in the month of delivery (Glovak et al., 2020). The use of opioids in pregnancy is rising, resulting in a growing number of infants diagnosed with neonatal opioid withdrawal syndrome, or neonatal abstinence syndrome, which impacts the CNS, autonomic nervous system, and gastrointestinal systems (McQueen and Murphy-Oikonen, 2016). Opioids pass through the placenta and might inhibit the rhythmic respiratory function in neonates (Hauser and Knapp, 2018). Paternal, maternal, and parental exposure to morphine during the adolescent developmental stage has been documented to provoke an escalation in anxiety- and depressive-like behaviors in the offspring (Odegaard et al., 2021).

Betaine constitutes a significant natural element found in nutrient-dense food sources and is frequently utilized as a dietary supplement. Beyond its role as an osmolyte and a methyl donor, betaine modulates the metabolism of lipids, sugars, ethanol, and homocysteine, exhibits properties associated with antioxidant and anti-inflammatory activities and alleviates endoplasmic reticulum stress and apoptotic processes (Lin et al., 2016). Furthermore, betaine demonstrates a variety of neuropharmacological effects, including anti-seizure properties, antidepressant-like effects and enhancements in memory function (Chen et al., 2021).

Neurodevelopmental reflexes are automatic and repetitive actions. This illustrates the brain stem and spinal cord reflexes utilized in newborns and infants. Changes in brain development result in atypical synaptogenesis, functioning, and myelination, leading to delays or absences in neurodevelopmental reflexes. Neurodevelopmental reflex assessment provides researchers with a testing approach that is not accessible in such young animals. The collection of reflexes mentioned encompasses, but is not confined to, limb grabbing and positioning, cliff evasion, righting, enhanced righting, walking, auditory surprise, posture, and eye opening. Abnormal reflexes result in delayed learning, missing responses, or their return later in life and are indicative of developmental disabilities. Therefore, experimental models of neurodevelopmental disorders can be utilized to assess reflex delays, and rodents serve as the perfect animal model to replicate these motor skills. Thus, this study sought to assess the protective impacts of betaine during pregnancy on postpartum depression, reflexive motor behaviors, and antioxidant levels in the offspring of mice experiencing morphine withdrawal syndrome.

## 2 Material and methods

### 2.1 Animals and experimental procedures

Animal experiments conducted in this study received approval from the Animal Ethics Committee of Razi University and adhered to the Guidelines for the Care and Use of Laboratory Animals in Research. A total of 14 male NMRI mice and 56 virgin female NMRI mice (weighing 28–30 g and aged 8–10 weeks) were sourced from the Pasture Institute in Tehran, Iran. The animals were kept in standard plastic cages (8 mice per cage) that were maintained under standard laboratory conditions with a temperature of 22 ± 2 °C and a 12-h light/12-h dark cycle, along with a standard pelleted chow diet (Pars Dam Co, Tehran, Iran) and fresh water available ad libitum. The animals were adapted for 1 week prior to starting the study. The first group served as the control group, consisting of male and female mice that did not receive morphine. In the second group, female mice received morphine for 21 days, whereas the male mice stayed abstinent from morphine. In the third group, male mice were exposed to morphine for 21 days, whereas the females remained abstinent from morphine. The fourth group included male and female mice that received morphine for 21 days. Eight female mice were given morphine for 21 days, and the females had no prior experience with drugs. The female mice not previously exposed to drugs were given betaine (30 mg/kg, Sigma Co. CAS No.: 590-47-6) during gestational days (GD) 5, 8, 11, 14, and 17. In group six, male mice were administered morphine for 21 days, and these males had not been exposed to the drug before. However, the women in this group had refrained from using morphine. The male mice without drugs were given betaine at a dose of 10 mg/kg on days 5, 8, 11, 14, and 17. In group 7, both male and female mice, which had no prior exposure to drugs, were given morphine for a duration of 21 days. Betaine (30 mg/kg) was administered to drug-naïve females on gestation days 5, 8, 11, 14, and 17. Male mice were administered betaine (30 mg/kg) on the same days. After the mice gave birth, pups from every litter (10 males) were chosen according to the distance from the anus to the vagina. These puppies were subsequently utilized to evaluate postnatal (PD) reflexes and neonatal motor activities. To avoid problems associated with body temperature and hunger, young mice were briefly kept away from their mother for a maximum of 15 min (Feather-Schussler and Ferguson, 2016).

### 2.2 Behavioral testing

#### 2.2.1 Ambulation

Young mice move by crawling until they hit postnatal day 5, after which they usually begin walking somewhere between 5 and 10 days old. An ambulation test was conducted to record this transitional phase when the mice were eight days old. The mice were positioned in a clear container visible from every angle during the experiment, and they were encouraged to move by lightly prodding their tails. A scoring system evaluated their mobility, with 0 indicating no movement, 1 for crawling with unsteady limb movement, 2 for slow crawling with steady limb movement, and 3 for rapid crawling or walking. To lessen the influence of learning, the assessment was performed three times within a 3-min interval (Morelli et al., 2011).

#### 2.2.2 Surface righting

A testing approach was utilized to assess the surface righting reflex, which involves the capability of young animals to revert to an upright position from a prone state. The experiment entailed positioning 5-7-day-old pups on their backs on a cotton sheet and recording the duration it took for them to flip back to their stomach after being released for 5 s. The test was administered three times in 3 min to verify precision, as there was no evidence of a persistent learning effect (Feather-Schussler and Ferguson, 2016).

#### 2.2.3 Hindlimb foot angle

This research focused on evaluating 8-day-old PD pups as they transitioned from crawling to walking. While walking, a significant change in the placement of the hind limbs was noted as they moved beneath the body. Feather-Schussler and Ferguson (2016) discovered that the angle formed by the hind limbs during walking posture was smaller than in the crawling position. A simple open space box, fitted with an overhead camera, monitored the movements of the puppies. The researchers encouraged the puppies to walk by gently tapping their tails. The foot angle was later evaluated by reviewing the captured videos, using a line from the heel/shin endpoint to the tip of the toe. Data were gathered solely from pups that completed a full stride in a straight path with their paws flat on the ground. To reduce experimental errors, the mean foot angles from three to five sets for every pup were calculated. It is important to mention that the experiment showed no significant learning linked to repetition (Feather-Schussler and Ferguson, 2016).

#### 2.2.4 Hindlimb suspension

The experiment was conducted when the pups were 12 to 15 days old, during which they could grasp the wire using both of their forelimbs. The puppies were freed after gripping the wire, and the duration until they fell was measured in seconds. The test was performed three times over 3 min to guarantee reliability and account for learning effects (Feather-Schussler and Ferguson, 2016).

#### 2.2.5 Grip strength

In this experiment, we evaluated the grip strength of PD7-8 offspring by monitoring their actions while holding onto a screen. For this purpose, a fiberglass screen measuring 16 × 18 was gradually tilted from horizontal to vertical to assess the four-limb grasping behavior of the animals (Kray et al., 2021).

#### 2.2.6 Front limb suspension

The experiment occurred when the pups were between 12 and 15 days old, during which they had the opportunity to grasp the wire with both forelimbs. The puppies were freed after gripping the wire, and the time taken for them to fall was noted in seconds. The assessment was performed three times over 3 min to ensure accuracy and account for learning effects (Feather-Schussler and Ferguson, 2016).

#### 2.2.7 Negative geotaxis

This research included conducting tests on the young of PD 7 mice. The puppies were placed on a hardwood floor slanted at a 45-degree angle and given the freedom to explore. In response to gravity vestibular signals, the researchers measured how long the pups rotated their bodies and oriented themselves with an upward slope (Feather-Schussler and Ferguson, 2016).

#### 2.2.8 Tail suspension test

TST was performed following the methodological guidelines set by Steru et al. (1985) and Can et al. (2012). Employing the same approach, the participants were situated far away from nearby stimuli and were placed under both sound and visual isolation to avoid any observation or interaction between them. The mice were raised 50 cm above the ground using adhesive tape attached about 1 cm from the tail's end, preventing them from escaping or attaching to nearby surfaces. The length of time without movement was carefully recorded during a 6-min period. Mice were considered immobile only if they showed no significant body shaking and did not move any limbs while being held in a fully passive and still position.

#### 2.2.9 Forced swimming test

FST was performed following the protocol outlined earlier in mouse models (Castagné et al., 2010). In this experimental setup, each mouse was separately immersed in a glass cylinder (height: 25 cm; diameter: 15 cm) filled with 10 cm of water kept at a temperature of 25 ± 1 °C for a period of 15 min (pre-test session). Twenty-four hours after the first exposure, the mouse was placed back in the cylinder and allowed to stay for a 6-min period (test session). A mouse was deemed immobile when it stopped resisting and stayed floating in a fixed position in the water, making only slight movements needed to keep its head above the surface. The total time spent immobile during the last 4 min of the 6-min testing period was documented and examined.

#### 2.2.10 Biochemical analysis

At the conclusion of the study, blood samples were collected from the mother mice, and serum levels of MDA, SOD, GPx, and CAT were measured using assay kits. Additionally, brain tissue from the offspring was collected, and levels of MDA, SOD, GPx, and CAT were measured.

### 2.3 Statistical analysis

The collected data were examined using one-way analysis of variance (ANOVA) and reported as mean ± standard error (SE). For treatments displaying notable differences, average values were compared utilizing the Tukey HSD test. Treatments with P-values under 0.05 were viewed as having meaningful differences.

## 3 Results

According to Fig. 1, the ambulation score significantly declined in offspring mice from the dam experiencing morphine withdrawal syndrome compared to the control group (P < 0.05). Moreover, the ambulation score notably dropped in the offspring of both male and female mice with morphine withdrawal syndrome relative to the control group (P < 0.05). The ambulation score in mice offspring of dams experiencing morphine withdrawal syndrome + betaine showed a significant improvement (P < 0.05). In mice offspring from males and females with morphine withdrawal syndrome + betaine group, ambulation score showed improvement (P < 0.05).

**Fig. 1:**
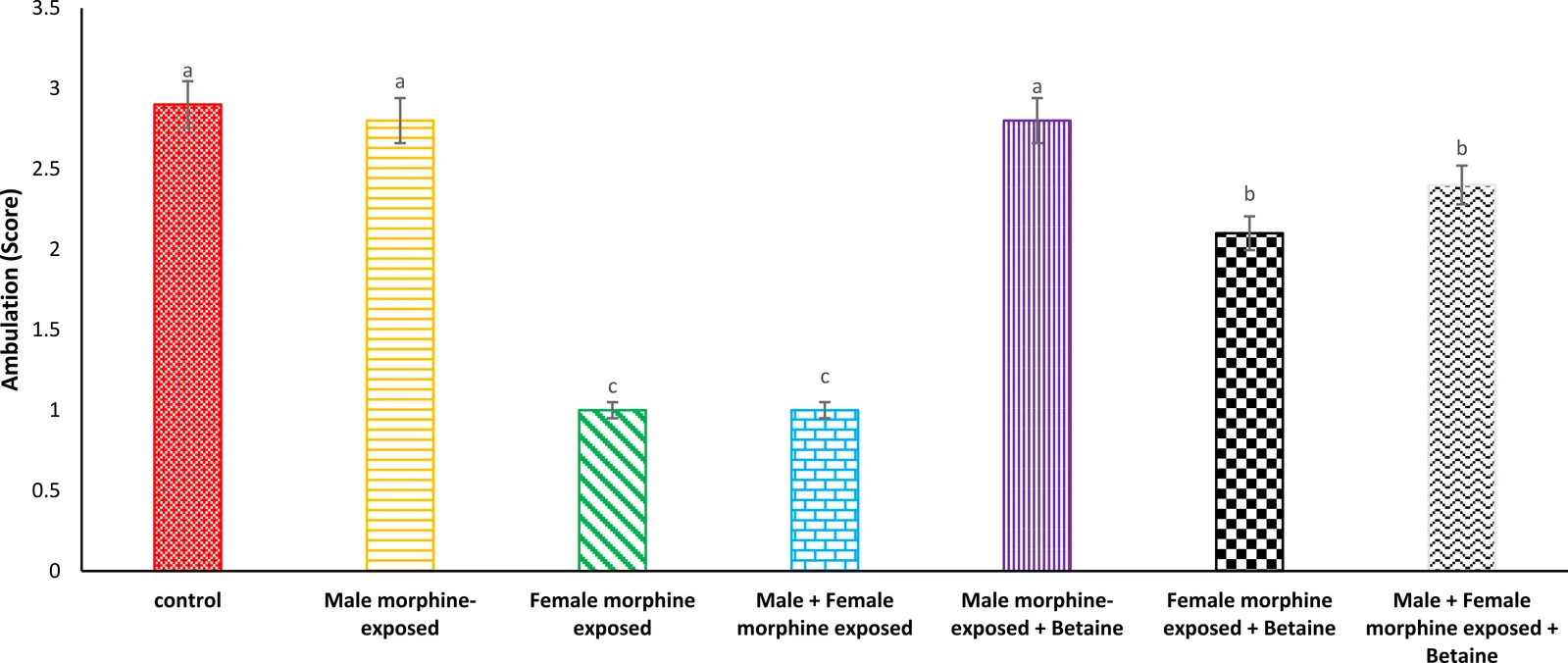
Effects of the betaine administration during pregnancy in parental with morphine withdrawal on ambulation score in mice offspring. Different letters (a, b and c) indicate significant differences for each group (P < 0.05). Data are presented as mean ± SE. Drug-naïve male injected with betaine (30 mg/kg) on days 5, 8, 11, 14 and 17 days. Drug-naïve female injected with betaine (30 mg/kg) on days 5, 8, 11, 14 and 17 days of gestation. Data are expressed as mean ± SE.

As demonstrated in Fig. 2, surface righting markedly rose in the offspring of the dam with morphine withdrawal syndrome compared to the control group (P < 0.05). Additionally, surface righting markedly improved in offspring of both male and female mice with morphine withdrawal syndrome when compared to the control group (P < 0.05). Surface righting in mouse offspring from dams experiencing morphine withdrawal syndrome and betaine showed significant improvement (P < 0.05). Surface righting improved in offspring mice from male and female with morphine withdrawal syndrome + betaine treatment (P < 0.05).

**Fig. 2:**
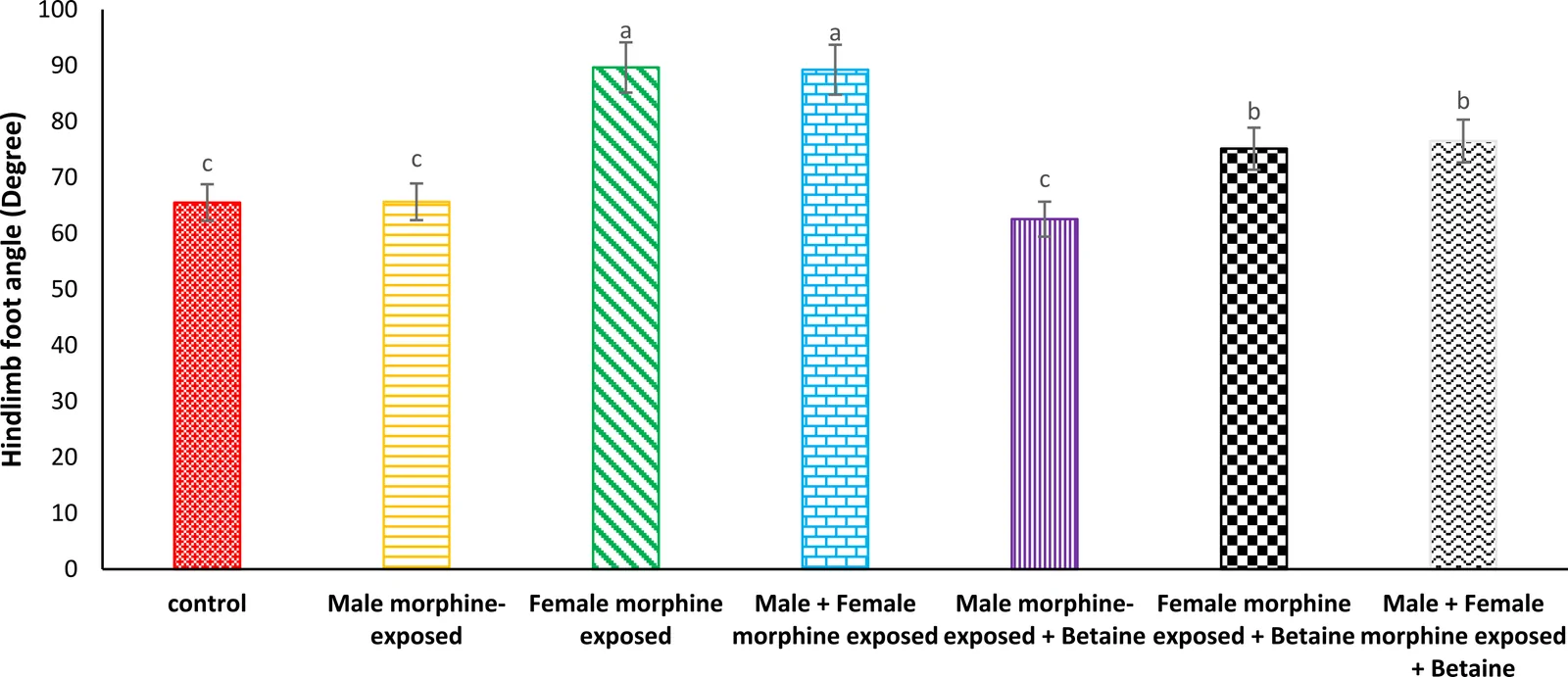
Effects of the betaine administration during pregnancy in parental with morphine withdrawal on hindlimb foot angle in mice offspring. Different letters (a, b and c) indicate significant differences for each group (P < 0.05). Data are presented as mean ± SE. Drug-naïve male injected with betaine (30 mg/kg) on days 5, 8, 11, 14 and 17 days. Drug-naïve female injected with betaine (30 mg/kg) on days 5, 8, 11, 14 and 17 days of gestation. Data are expressed as mean ± SE.

Fig. 3 shows that the hindlimb foot angle was notably higher in the mice offspring from the dam with morphine withdrawal syndrome when compared to the control group (P < 0.05). Likewise, hindlimb foot angle was greater in offspring of male and female mice with morphine withdrawal syndrome than in the control group (P < 0.05). As observed, hindlimb foot angle showed improvement in offspring of dams with morphine withdrawal syndrome + betaine (P < 0.05). In mice offspring from males and females with morphine withdrawal syndrome + betaine group, hindlimb foot angle showed improvement (P < 0.05).

**Fig. 3:**
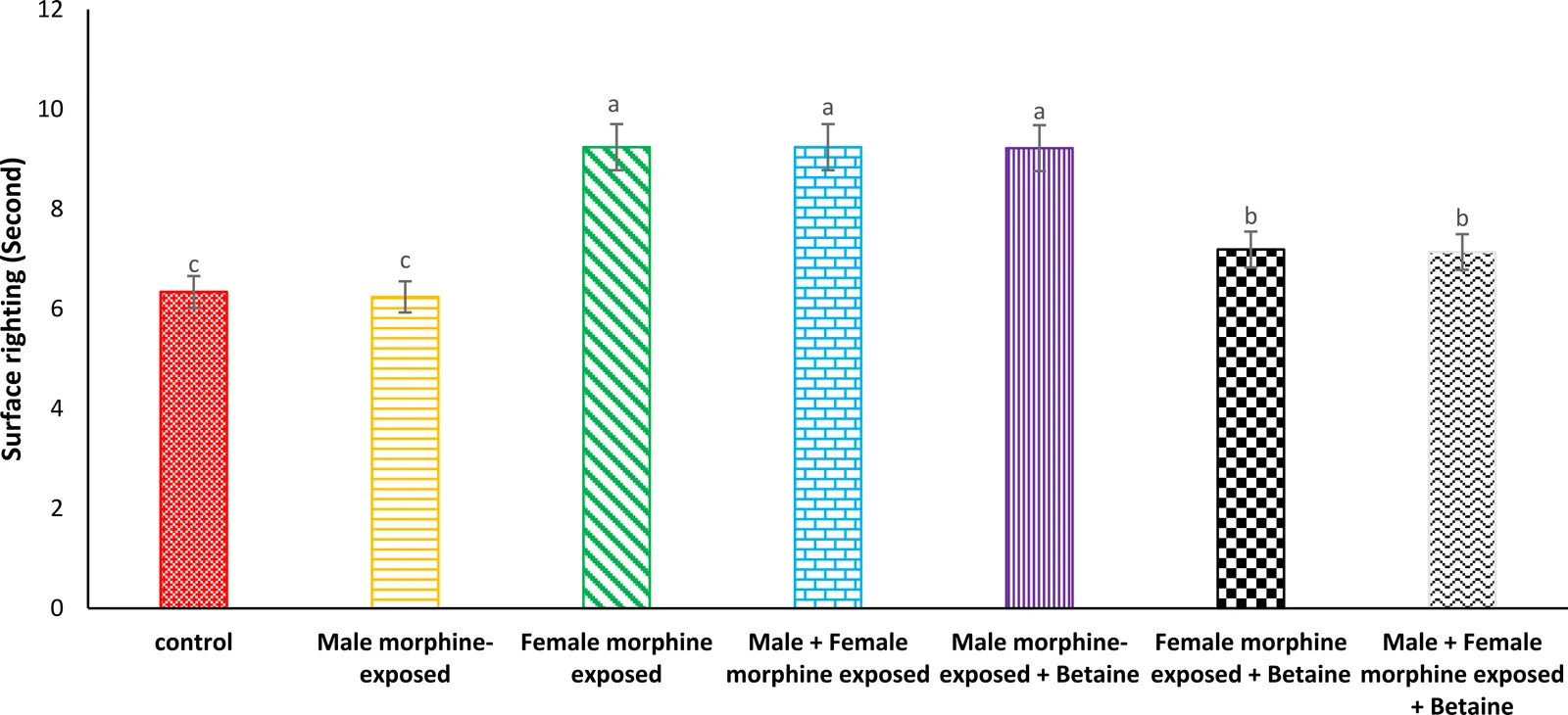
Effects of the betaine administration during pregnancy in parental with morphine withdrawal on surface righting in mice offspring. Different letters (a, b and c) indicate significant differences for each group (P < 0.05). Data are presented as mean ± SE. Drug-naïve male injected with betaine (30 mg/kg) on days 5, 8, 11, 14 and 17 days. Drug-naïve female injected with betaine (30 mg/kg) on days 5, 8, 11, 14 and 17 days of gestation. Data are expressed as mean ± SE.

As shown in Fig. 4, hindlimb suspension was significantly reduced in the offspring of mice from the morphine withdrawal syndrome group compared to the control group (P < 0.05). Moreover, hindlimb suspension was notably reduced in offspring from male and female mice exhibiting morphine withdrawal syndrome when compared to the control group (P < 0.05). Hindlimb suspension enhanced in the offspring of dams with morphine withdrawal syndrome + betaine (P < 0.05). Hindlimb suspension showed improvement in progeny of males and females experiencing morphine withdrawal syndrome in the betaine group (P < 0.05).

**Fig. 4:**
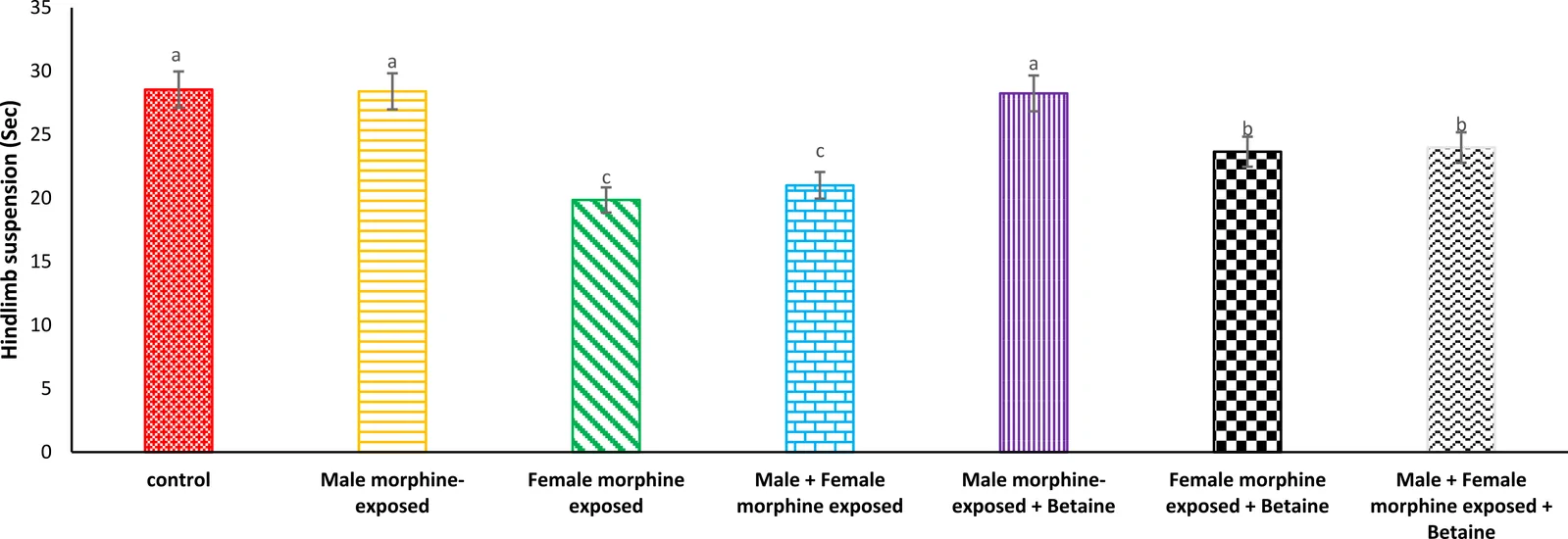
Effects of the betaine administration during pregnancy in parental with morphine withdrawal on hindlimb suspension in mice offspring. Different letters (a, b and c) indicate significant differences for each group (P < 0.05). Data are presented as mean ± SE. Drug-naïve male injected with betaine (30 mg/kg) on days 5, 8, 11, 14 and 17 days. Drug-naïve female injected with betaine (30 mg/kg) on days 5, 8, 11, 14 and 17 days of gestation. Data are expressed as mean ± SE.

According to Fig. 5, grip strength noticeably diminished in the offspring of dams experiencing morphine withdrawal syndrome in comparison to the control group (P < 0.05). Furthermore, grip strength was notably lower in the offspring of both male and female mice with morphine withdrawal syndrome compared to the control group (P < 0.05). Grip strength in mice offspring from dams with morphine withdrawal syndrome + betaine showed significant improvement (P < 0.05). Grip strength increased in the offspring of male and female mice with morphine withdrawal syndrome + betaine group (P < 0.05).

**Fig. 5:**
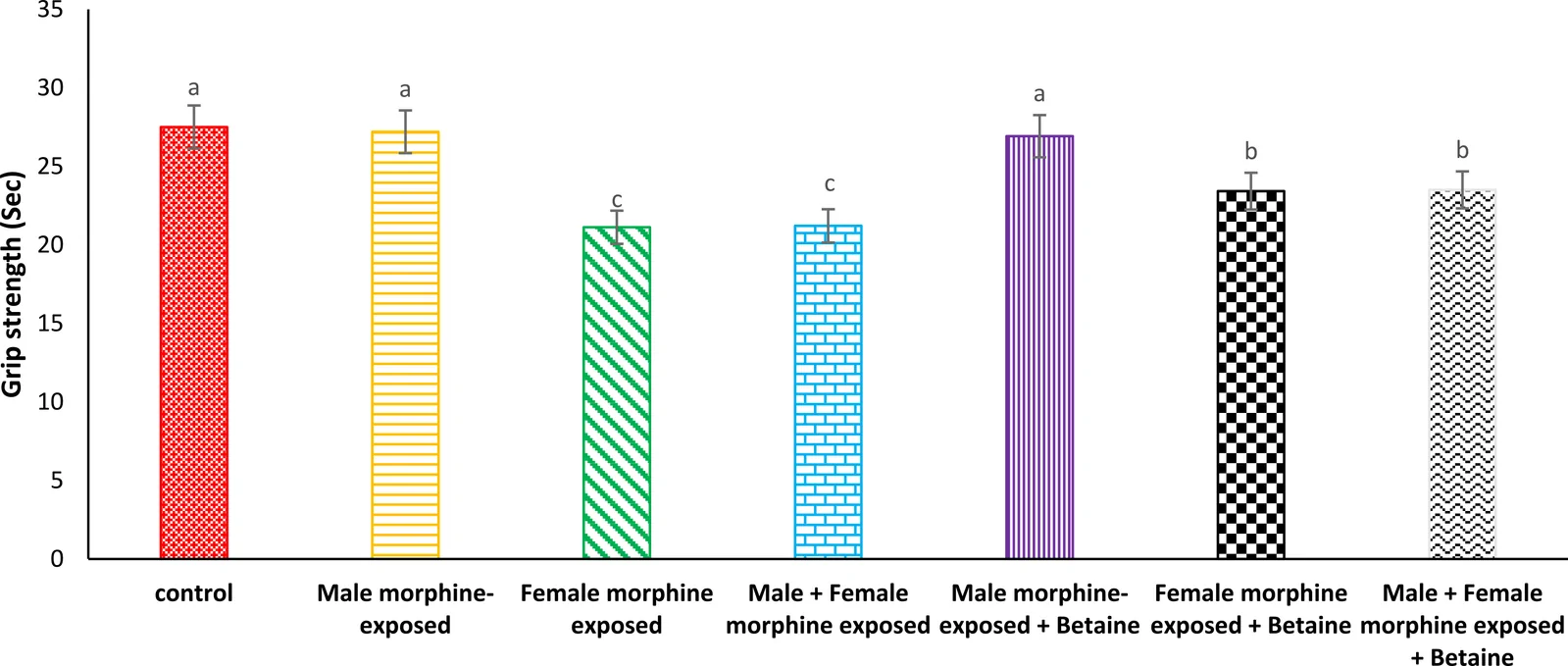
Effects of the betaine administration during pregnancy in parental with morphine withdrawal on grip strength in mice offspring. Different letters (a, b and c) indicate significant differences for each group (P < 0.05). Data are presented as mean ± SE. Drug-naïve male injected with betaine (30 mg/kg) on days 5, 8, 11, 14 and 17 days. Drug-naïve female injected with betaine (30 mg/kg) on days 5, 8, 11, 14 and 17 days of gestation. Data are expressed as mean ± SE.

As depicted in Fig. 6, front limb suspension notably diminished in the offspring of mice from the morphine withdrawal syndrome group compared to the control group (P < 0.05). Furthermore, the front limb suspension notably reduced in the offspring of both male and female mice with morphine withdrawal syndrome compared to the control group (P < 0.05). Front limb suspension was enhanced in mice offspring from dams with morphine withdrawal syndrome + betaine (P < 0.05). Front limb suspension enhancement was observed in mice offspring from both male and female with morphine withdrawal syndrome + betaine group (P < 0.05).

**Fig. 6:**
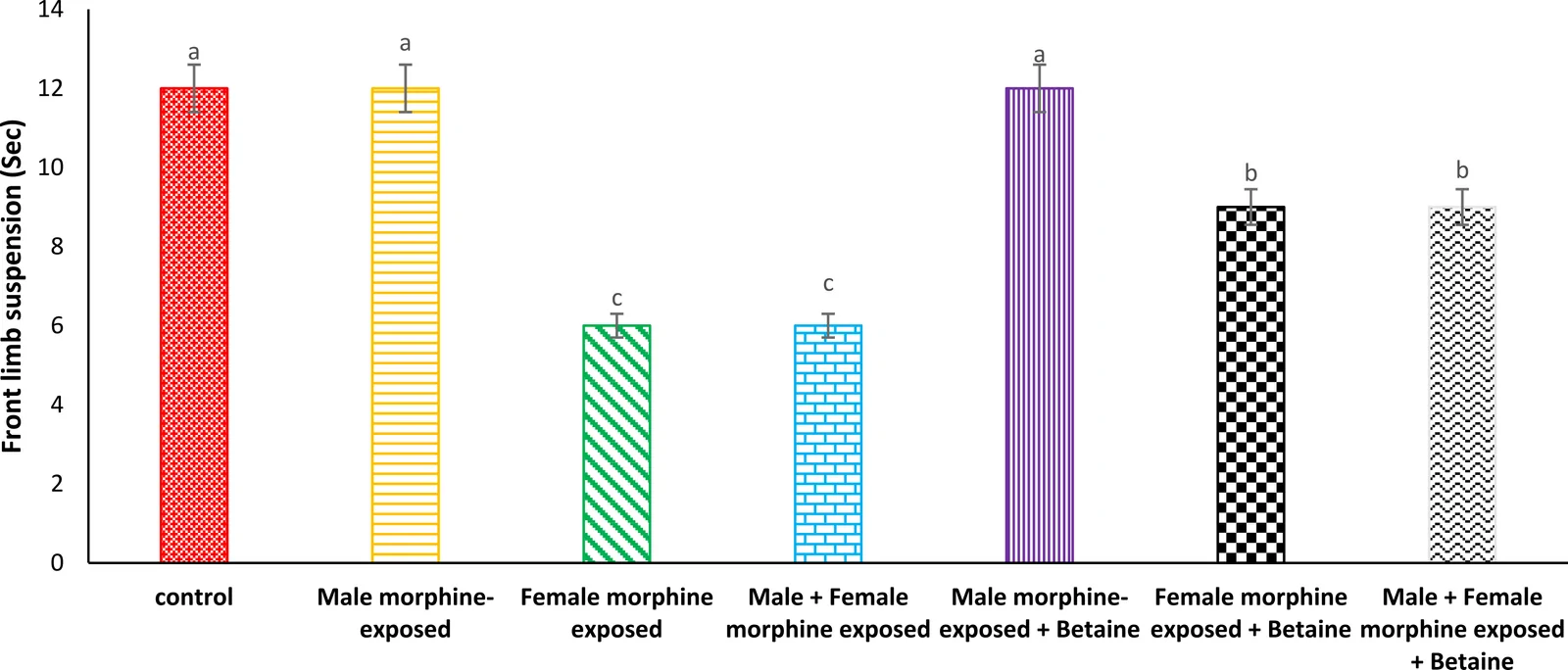
Effects of the betaine administration during pregnancy in parental with morphine withdrawal on front limb suspension in mice offspring. Different letters (a, b and c) indicate significant differences for each group (P < 0.05). Data are presented as mean ± SE. Drug-naïve male injected with betaine (30 mg/kg) on days 5, 8, 11, 14 and 17 days. Drug-naïve female injected with betaine (30 mg/kg) on days 5, 8, 11, 14 and 17 days of gestation. Data are expressed as mean ± SE.

Fig. 7 shows that negative geotaxis was significantly greater in the offspring of dams from the morphine withdrawal syndrome group compared to the control group (P < 0.05). Likewise, negative geotaxis was higher in the offspring of both male and female mice with morphine withdrawal syndrome compared to the control group (P < 0.05). Negative geotaxis enhancement was observed in offspring mice from dams experiencing morphine withdrawal syndrome + betaine (P < 0.05). Negative geotaxis enhanced in offspring of both male and female experiencing morphine withdrawal syndrome + betaine group (P < 0.05).

**Fig. 7:**
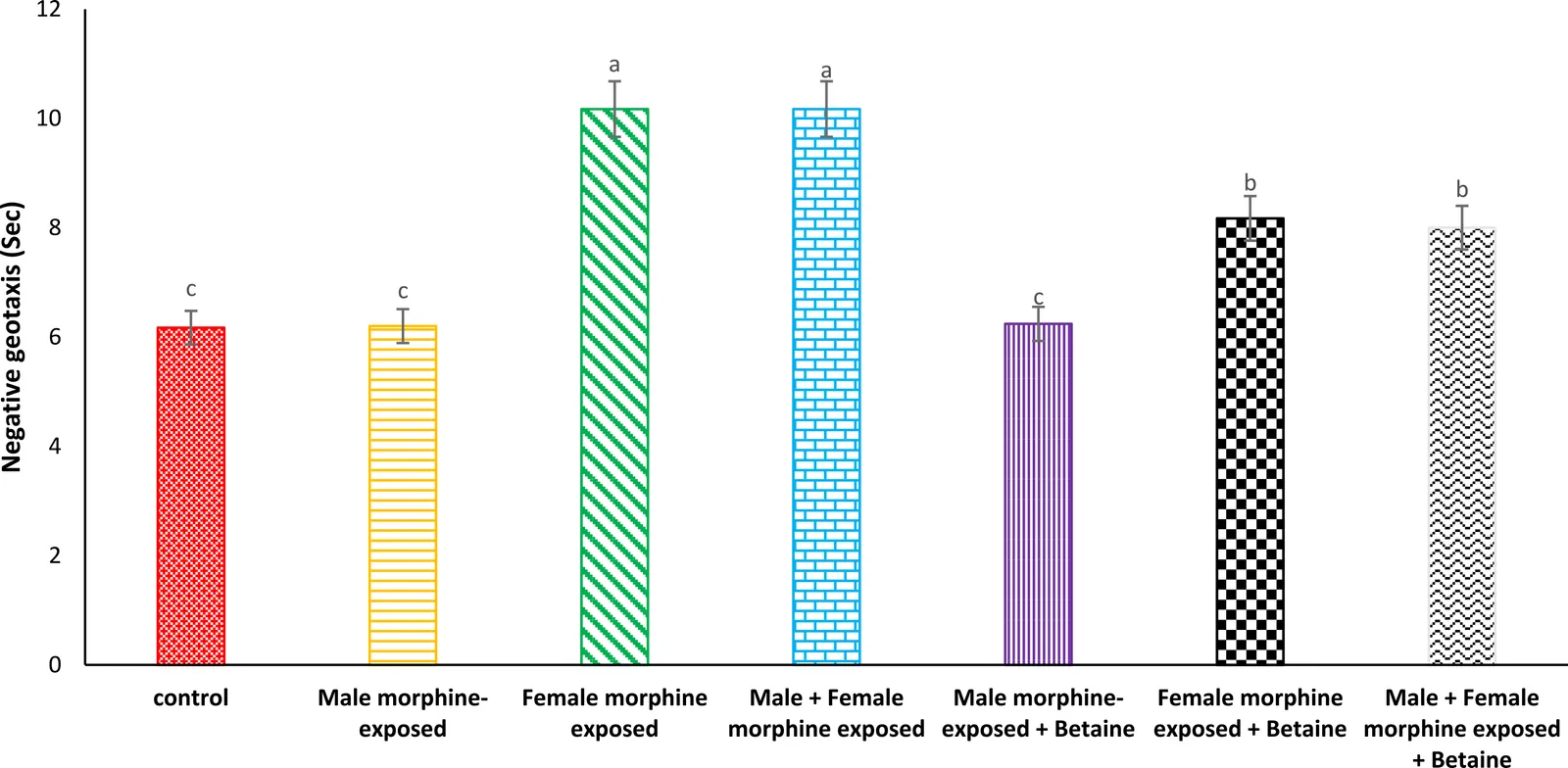
Effects of the betaine administration during pregnancy in parental with morphine withdrawal on negative geotaxis in mice offspring. Different letters (a, b and c) indicate significant differences for each group (P < 0.05). Data are presented as mean ± SE. Drug-naïve male injected with betaine (30 mg/kg) on days 5, 8, 11, 14 and 17 days. Drug-naïve female injected with betaine (30 mg/kg) on days 5, 8, 11, 14 and 17 days of gestation. Data are expressed as mean ± SE.

Based on Fig. 8, the duration of immobility measured by TST markedly rose in mice experiencing morphine withdrawal syndrome after delivery when compared to the control group (P < 0.05). Moreover, the duration of immobility recorded in the TST showed significant improvement in mice experiencing morphine withdrawal syndrome post-delivery when compared to the control group (P < 0.05). Moreover, the duration of immobility measured by TST showed a notable improvement in mice with morphine withdrawal syndrome + betaine post-delivery when compared to the control group (P < 0.05).

**Fig. 8:**
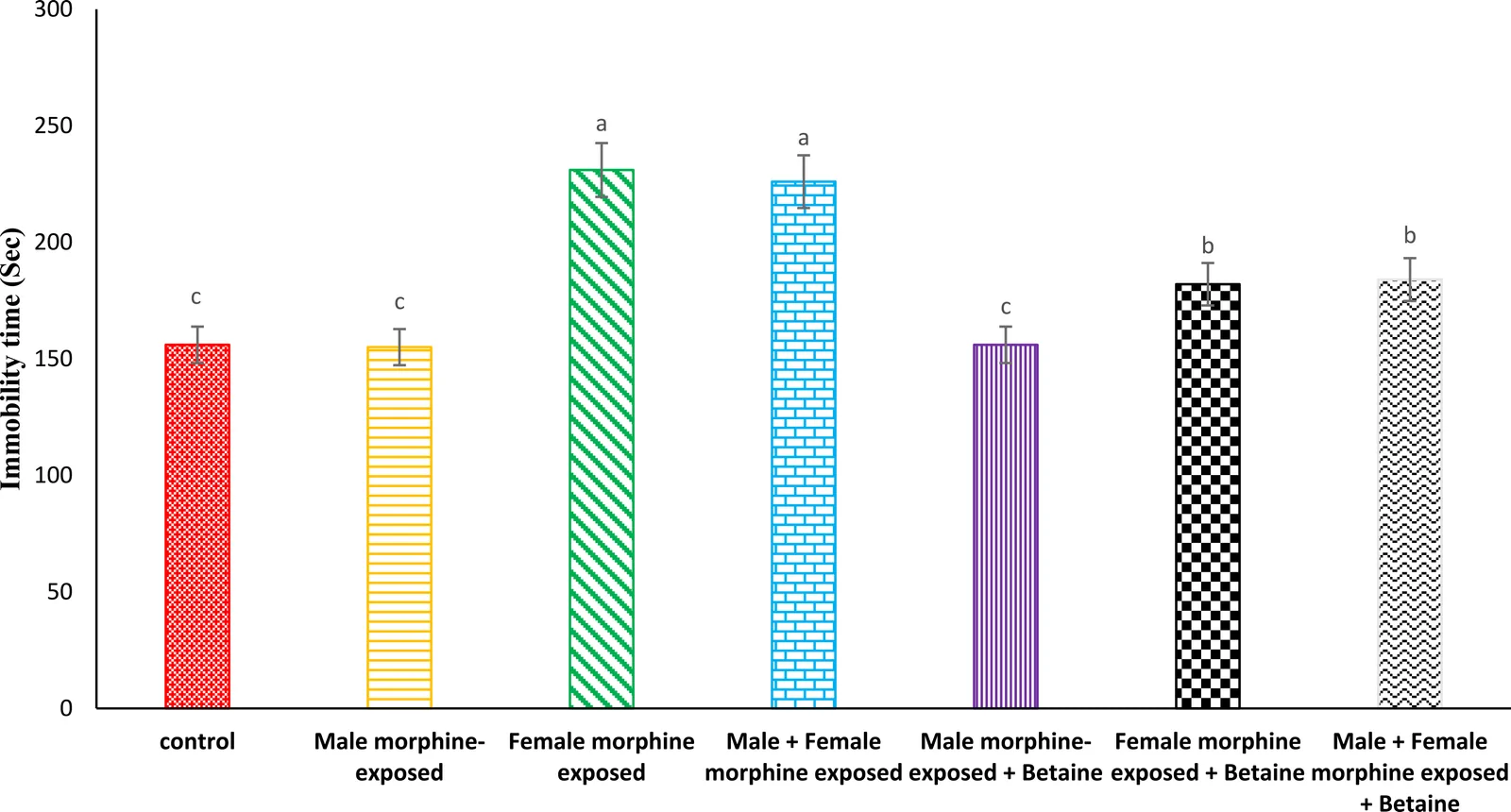
Effects of the betaine administration during pregnancy in parental with morphine withdrawal on immobility time using TST in mice offspring. Different letters (a, b and c) indicate significant differences for each group (P < 0.05). Data are presented as mean ± SE. Drug-naïve male injected with betaine (30 mg/kg) on days 5, 8, 11, 14 and 17 days. Drug-naïve female injected with betaine (30 mg/kg) on days 5, 8, 11, 14 and 17 days of gestation. Data are expressed as mean ± SE.

Fig. 9 indicates that the immobility duration in the FST significantly rose in mice experiencing morphine withdrawal syndrome post-delivery when compared to the control group (P < 0.05). Moreover, the time spent immobile in the FST showed a significant improvement in mice experiencing morphine withdrawal syndrome after delivery when compared to the control group (P < 0.05). Moreover, the duration of immobility in the FST notably increased in mice experiencing morphine withdrawal syndrome + betaine after administration compared to the control group (P < 0.05).

**Fig. 9:**
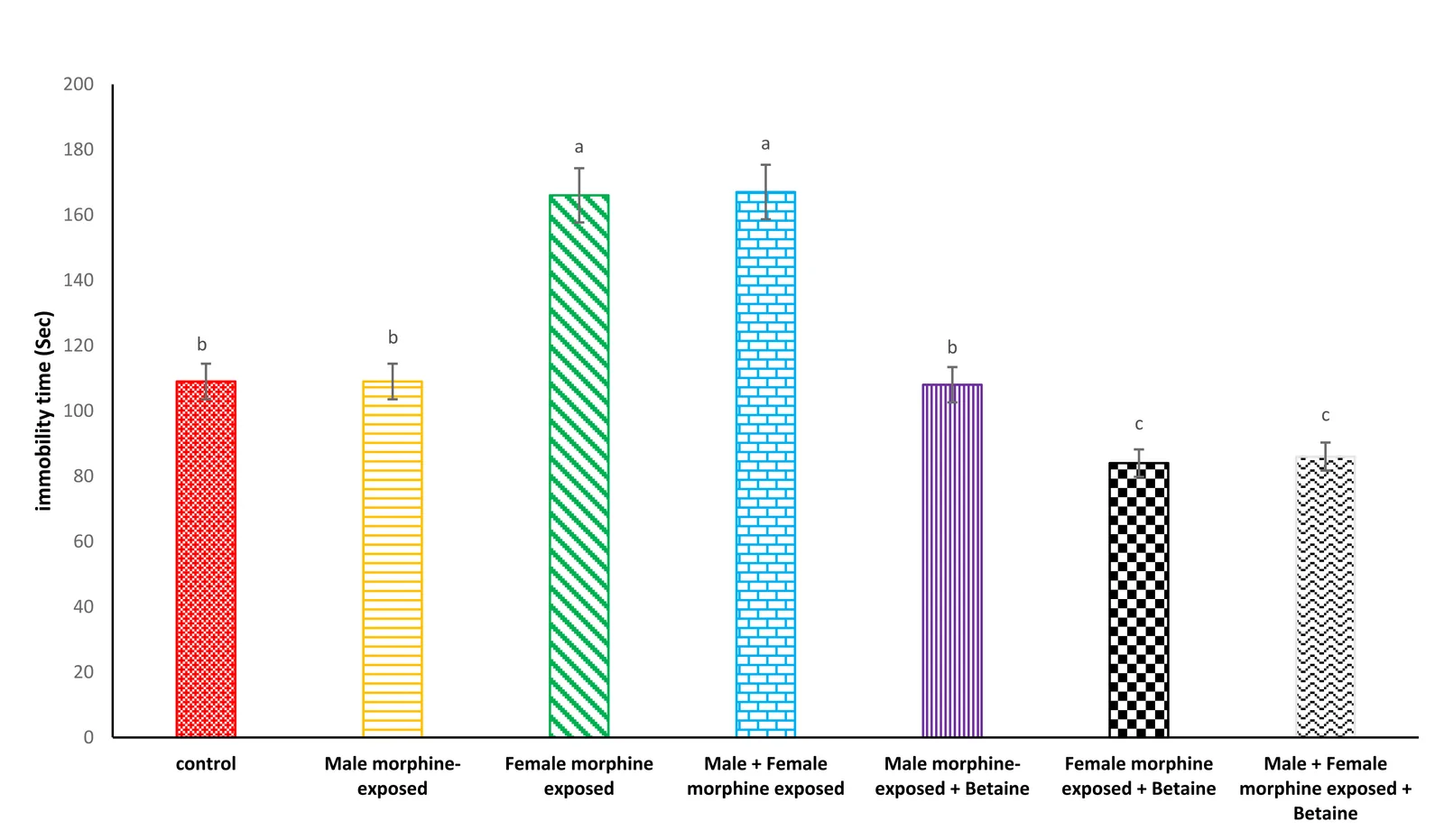
Effects of the betaine administration during pregnancy in parental with morphine withdrawal on immobility time using FST in mice offspring. Different letters (a, b and c) indicate significant differences for each group (P < 0.05). Data are presented as mean ± SE. Drug-naïve male injected with betaine (30 mg/kg) on days 5, 8, 11, 14 and 17 days. Drug-naïve female injected with betaine (30 mg/kg) on days 5, 8, 11, 14 and 17 days of gestation. Data are expressed as mean ± SE.

Fig. 10 indicates that the number of cross in the OFT significantly decreased in mice experiencing morphine withdrawal syndrome post-delivery when compared to the control group (P < 0.05). Moreover, number of cross in the OFT notably increased in mice experiencing morphine withdrawal syndrome + betaine after administration compared to the control group (P < 0.05).

**Fig. 10:**
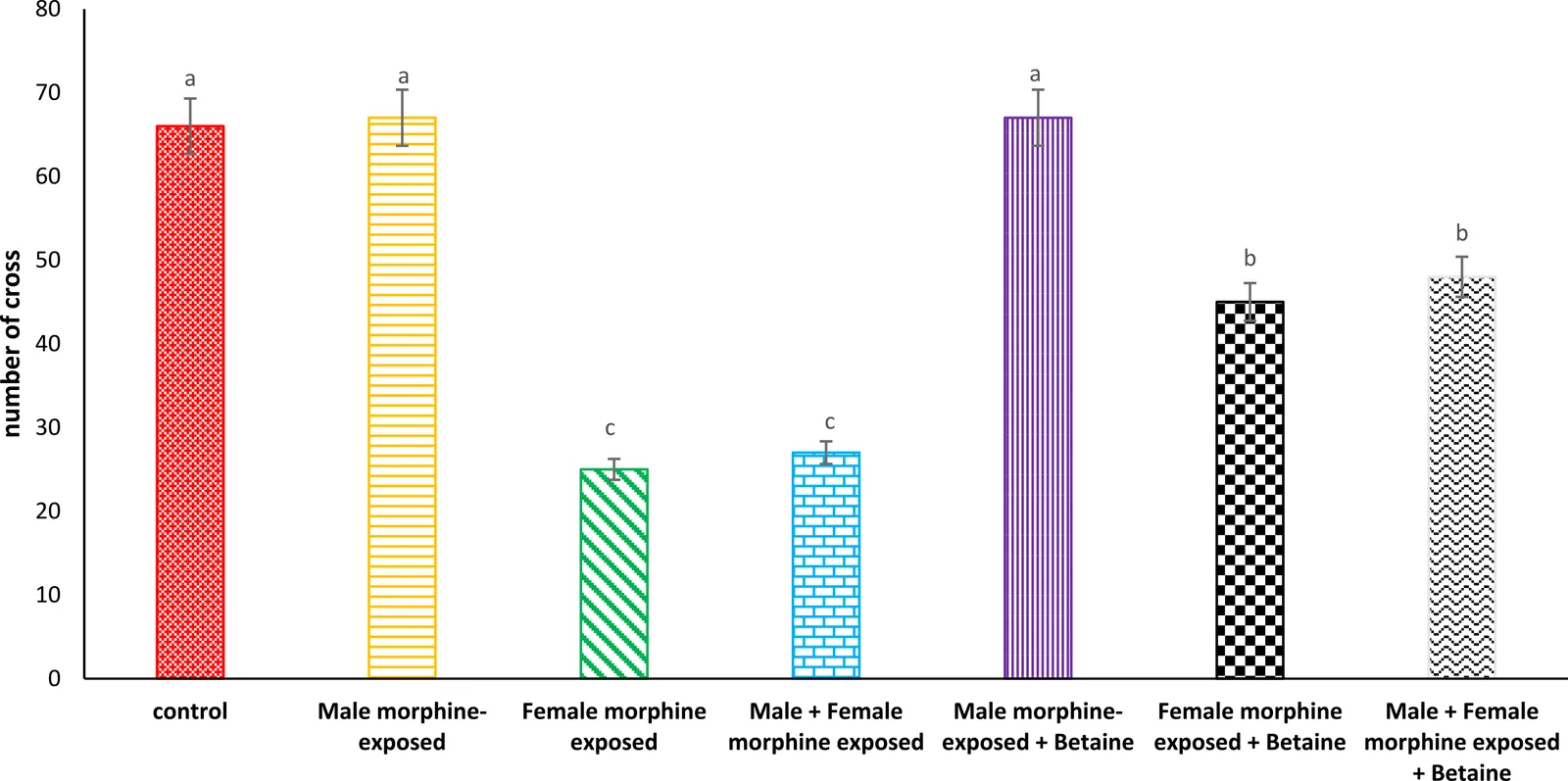
Effects of the betaine administration during pregnancy in parental with morphine withdrawal on number of cross using OFT in mice offspring. Different letters (a, b and c) indicate significant differences for each group (P < 0.05). Data are presented as mean ± SE. Drug-naïve male injected with betaine (30 mg/kg) on days 5, 8, 11, 14 and 17 days. Drug-naïve female injected with betaine (30 mg/kg) on days 5, 8, 11, 14 and 17 days of gestation. Data are expressed as mean ± SE.

Morphine administration increased MDA and decreased SOD, GPx and CAT levels in serum of the mothers compared to control group (P < 0.05). The administration of betaine decreased MDA and enhanced SOD, GPx and CAT levels in serum of the mothers compared to control group (P < 0.05) (Table 1).

**Table 1:** Effects of the Betaine administration during pregnancy in parental with morphine withdrawal on serum antioxidant levels in post-partum mice.

	MDA (nmol/ml)	SOD (IU/ml)	GPx (nmol/ml)	CAT (nmol/ml)
**Control**	43.34 ± 2.45^c^	20.11 ± 1.45^a^	12.25 ± 1.42^a^	16.24 ± 1.10^a^
**Male morphine-exposed**	43.57 ± 2.68^c^	20.27 ± 1.57^a^	12.09 ± 1.14^a^	16.68 ± 1.14^a^
**Female morphine exposed**	62.72 ± 2.94^a^	13.13 ± 1.43^c^	6.42 ± 1.32^c^	10.35 ± 1.13^c^
**Male** **+** **Female morphine-exposed**	63.45 ± 2.21^a^	13.67 ± 1.23^c^	5.87 ± 1.97^c^	11.45 ± 1.98^c^
**Male morphine-exposed** **+** **Betaine**	42.28 ± 2.32^c^	21.23 ± 1.87^a^	12.77 ± 1.15^a^	16.70 ± 1.17^a^
**Female morphine exposed** **+** **Betaine**	56.33 ± 2.65^b^	15.43 ± 1.63^b^	9.54 ± 1.17^b^	12.24 ± 1.21^b^
**Male** **+** **Female morphine exposed** **+** **Betaine**	51.19 ± 2.12^b^	15.76 ± 1.19^b^	10.24 ± 1.09^b^	12.25 ± 1.14^b^

MDA: malondialdehyde, SOD: superoxide dismutase, GPx: glutathione peroxidase, CAT: catalase. Different letters (a, b and c) indicate significant differences for each group in each estrous cycle phase (P < 0.05). Data are presented as mean ± SE. Drug-naïve male injected with Betaine (30 mg/kg) on days 5, 8, 11, 14 and 17 days. Drug-naïve female injected with Betaine (30 mg/kg) on days 5, 8, 11, 14 and 17 days of gestation. Data are expressed as mean ± SE.

Also, morphine administration increased MDA and decreased SOD, GPx and CAT levels in brain tissue of the offspring compared to control group (P < 0.05). The administration of betaine decreased MDA and enhanced SOD, GPx and CAT levels in brain tissue of the offspring compared to control group (P < 0.05) (Table 2).

**Table 2:** Effects of the Betaine administration during pregnancy in parental with morphine withdrawal on brain tissue antioxidant levels in mice offspring.

	MDA (μg/mg protein)	SOD (μg/mg protein)	GPx (μg/mg protein)	CAT (μg/mg protein)
**Control**	12.26 ± 1.58^c^	4.12 ± 0.53^a^	3.66 ± 0.13^a^	0.96 ± 0.11^a^
**Male morphine-exposed**	12.10 ± 1.40^c^	4.24 ± 0.25^a^	3.58 ± 0.12^a^	0.98 ± 0.16^a^
**Female morphine exposed**	22.64 ± 1.42^a^	2.95 ± 0.37^c^	0.56 ± 0.11^c^	0.21 ± 0.11^c^
**Male** **+** **Female morphine exposed**	23.14 ± 1.12^a^	2.55 ± 0.67^c^	0.51 ± 0.16^c^	0.22 ± 0.16^c^
**Male morphine-exposed** **+** **Betaine**	12.67 ± 1.37^c^	4.12 ± 0.85^a^	3.97 ± 0.19^a^	0.94 ± 0.13^a^
**Female morphine exposed** **+** **Betaine**	18.99 ± 1.65^b^	1.99 ± 0.93^b^	2.04 ± 0.17^b^	0.62 ± 0.04^b^
**Male** **+** **Female morphine exposed** **+** **Betaine**	17.34 ± 1.99^b^	1.98 ± 0.90^b^	2.40 ± 0.10^b^	0.65 ± 0.05^b^

MDA: malondialdehyde, SOD: superoxide dismutase, GPx: glutathione peroxidase, CAT: catalase. Different letters (a, b and c) indicate significant differences for each group in each estrous cycle phase (P < 0.05). Data are presented as mean ± SE. Drug-naïve male injected with Betaine (30 mg/kg) on days 5, 8, 11, 14 and 17 days. Drug-naïve female injected with Betaine (30 mg/kg) on days 5, 8, 11, 14 and 17 days of gestation. Data are expressed as mean ± SE.

## 4 Discussion

Based on the literature, this is first report regarding on effects of the betaine supplementation during pregnancy on postpartum depression, reflexive motor behaviors and brain tissue antioxidant levels in mice offspring. Based on main findings, morphine treatment notably elevated post-partum immobility duration in TST and FST. Also, reflexive motor behaviors decreased following prenatal morphine exposure; however, no difference found between male mice exposed to morphine and female mice. The administration of morphine markedly increased MDA levels while SOD, GPx, and CAT levels in the serum of mothers and the brain tissue of the pups, respectively. Velíšek et al. (2003) report that exposure to morphine during prenatal development in rats causes stress conditions in both male and female subjects. Chronic morphine administration creates a condition of persistent stress in mothers, leading to hyperactivity in the hypothalamic-pituitary-adrenal (HPA) axis, which may alter the HPA axis development in offspring, resulting in excessive glucocorticoid release, recognized as significant contributors to psychiatric-like disorders, including anxiety and depression (Castro et al., 2021). Both male and female offspring subjected to high morphine doses in the perinatal period exhibit anxiety- and depression-like behaviors irrespective of sex in adulthood (Castro et al., 2021). Ahmadalipour et al. (2015) found that prenatal exposure to morphine from GD 11 to GD18 triggered anxiety-like behaviors in young rats. In contrast, the study by Tan et al. (2015) identified an anxiolytic effect in adult rats exposed to morphine in utero from GD9 to GD18, using the same dosage. Certainly, other studies have shown that prenatal exposure to such morphine doses can lead to depressive-like behaviors in rodents as a result of elevated catecholamines (noradrenaline) in the hypothalamus and decreased levels of corticosteroids in adult blood (Klausz et al., 2011). Noradrenergic pathway, influenced by the opioid system, plays a crucial role in neurodevelopment that begins during early pregnancy (GD10-13) and attains mature signaling by post-delivery day 70, various timing and durations of intervention may lead to distinct changes (Saboory et al., 2020). Unplanned pregnancies often happen among women dependent on opioids, leading to a high prevalence of both opioid maintenance and relapse risk. These theories of neuroendocrine pathophysiology necessitate additional research and represent the limitations of the current study. Tan et al. (2015) revealed a comparable outcome with males, where prenatal exposure to morphine (5 mg/kg followed by 10 mg/kg; GD9-18) did not influence the spontaneous locomotor behavior. Regrettably, Tan et al. (2015) study did not assess females. Moreover, the finding that the highest density of neuronal opioid receptors is found in the limbic system, thalamus, striatum, hypothalamus, midbrain, and spinal cord implies that physiological processes beyond analgesia and pain perception could be influenced by narcotics. Since opioid medications influence the opioid pathway in neurodevelopment, we opted to explore the pain threshold in both sexes (Castro et al., 2021; McDonough et al., 2024). In the nociception test, significant prenatal exposure to morphine likewise influenced the pain perception profile. Indeed, research indicates that there are distinctions between males and females in pain sensitivity, with females appearing to be more responsive to pain perception and showing greater negative reactions to nociceptive stimuli compared to males (Castro et al., 2021). Betaine intake was not related to psychological disorders (Kazeminejad et al., 2025). Betaine administration (50 and 100 mg/kg bw) orally uninterrupted for 28 days improved movement in open field, performed negative geotaxis, and a forelimb grip test in mice which finally enhanced locomotive, motor, and anxiolytic performance, increased the antioxidant signaling mechanisms and demurred oxidative, inflammatory, and apoptotic biomarkers and histoarchitectural damage in the cerebrum and cerebellum cortices mediated by sodium fluoride-induced neurotoxicity (Owumi et al., 2024a) and our findings on behavioral test was in agreement to this report. Betaine enhanced balance, motor coordination, and grasping ability, while decreasing oxidative stress, inhibiting interleukin (IL)-4 and IL-17 levels, and reversing the demyelination caused by cuprizone in a rat model of demyelination (Singhal et al., 2020; Essawy et al., 2025) and our findings on behavioral test was in agreement to this report.

In a recent study, it was revealed that dams administered morphine throughout gestation exhibited markedly reduced pup-directed behavior, increased nursing fragmentation, and elevated entropy scores. During adolescence, male and female rats that were exposed to morphine showed a decreased preference for sucrose and, as adults, engaged less in interactions with familiar peers. Variations in social behaviors were associated with heightened activity in non-dopaminergic cells within mesolimbic reward areas of the brain. Collectively, these findings indicate that exposure to morphine during the perinatal period fosters anhedonic behavior, likely because of inconsistent and erratic maternal behavior in opioid-dependent mothers (Searles et al., 2025). Our novel findings were in agreement to previous reports which prenatal exposure to morphine had adverse effect on reflexive motor behaviors in mice offspring's.

In the current research, administration of betaine and morphine considerably enhanced reflexive motor activities in mouse offspring. The administration of betaine did not produce any notable changes in reflexive motor behaviors among the male cohort exposed to morphine. Betaine treatment significantly reduced immobility duration in TST and FST. The administration of betaine decreased MDA and enhanced SOD, GPx and CAT levels in serum of the mothers and brain tissue of the pups. As a methyl donor, betaine has been considered an efficient antioxidant substance, whereas its mechanism by which betaine acts as an antioxidant remains unknown. Betaine may serve as an antioxidant by elevating the activities of SOD and CAT in serum. CAT, SOD, and GPx are key antioxidant enzymes in living organisms that can directly neutralize reactive oxygen and prevent additional harm (Zhao et al., 2018). SOD activity reflected the antioxidant potential in the testis. Additionally, betaine has demonstrated an ability to improve GPx activity in the intestines of weaned piglets. GPx is involved in glutathione metabolism, protecting the intracellular redox balance of glutathione to avert oxidative harm to cells (Han et al., 2017). MDA results from the oxidation reactions involving free radicals and lipids in cell membranes, indicating the levels of ROS in the body (Tsikas, 2017). Betaine is the substrate of the betaine homocysteinem methyltransferase (BHMT) reaction, which converts homocysteine to methionine (Li et al., 2022). In this reaction, a methyl group is transferred from betaine to homocysteine to form methionine (Alipourfard et al., 2023). Methionine plays an important role in the scavenging of ROS, which is particularly susceptible to ROS and can be oxidized to methionine sulfoxide by ROS. On the other hand, methionine sulfoxide could be reduced back to methionine-by-methionine sulfoxide reductases. Each methionine oxidation reduction cycle could destroy one equivalent of ROS, thus serving as a scavenging system for ROS (Li et al., 2022). Betaine induced oxidative stress along with anti-proliferative and apoptotic impacts as evidenced by the levels of antioxidant as well as apoptotic proteins (CASP3). Betaine supplementation can enhance testicular antioxidant capacity in Hu sheep. Collectively, these findings validated the antioxidant characteristics of betaine (Cai et al., 2021; Kret-Bułat et al., 2026). Betaine is synthesized in mitochondria from choline via choline dehydrogenase and actively transported into cells by organic osmolyte transporter betaine/γ-aminobutyric acid transporter. Mechanisms may interact with brain signaling pathways involved in the acquisition of physical dependence to opioids and expression of naloxone HCl-precipitated withdrawal, including pathways involving N-methyl D-aspartate glutamatergic receptors, muscarinic receptors, corticotropin releasing factor (CRF) receptor CRF1, tachykinin receptors and oxidative stress (Cai et al., 2021). Betaine administration (50 and 100 mg/kg bw) orally uninterrupted for 28 days improved nonenzymatic and enzymatic antioxidants (GSH GST, GPx, SOD, CAT) in the cerebrum and cerebellum cortices mediated by sodium fluoride-induced neurotoxicity (Owumi et al., 2024a) and our findings on behavioral test was in agreement to this report. This may be driven by the modulation of the Keap1-Nrf2-ARE signaling pathway as indicated by the strong binding affinity and interactions of betaine with Keap1-Nrf2 complex as indicated by in silico investigation as depicted in our molecular mechanisms of Chemico-biological interaction to ultimately confers cellular protection (Owumi et al., 2024b). It is worthy of note that, the in-silico analysis presented in the current study complements our previous result (Owumi et al., 2024b), which showed the binding tendency of betaine to human Keap1 protein.

Betaine reduced all naloxone HCl-induced behavioral and physical effects, suggesting that betaine may influence essential intracellular mechanisms crucial for the establishment of morphine physical dependence in rats. The preceding discussion is directly pertinent to possible mechanisms through which betaine reduced the development of morphine dependency (Li et al., 2021). Sneezing is a frequent symptom of opioid withdrawal in people as well as in laboratory animals. A wealth of information exists regarding the neural processes underlying sneezing (Ramirez et al., 2022), and it is now clear that the cellular actions triggered by betaine can be included among these possible mechanisms.

## 5 Conclusion

In conclusion these findings suggested maternal exposure to betaine seems to offer protection against the negative impacts of morphine on post-partum depression and reflexive motor behaviors in the mothers and pups.

## Author contribution

Negin-sadat Zargarian: data collection, draft of paper.

Shahin Hassanpour: supervisor, methodology, revise the paper.

Razieh Hosseini: advisor.

## Funding

None.

## Declaration of competing interest

None.

## Data Availability

No data was used for the research described in the article.
